# Sample Size and Geometric Morphometrics Methodology Impact the Evaluation of Morphological Variation

**DOI:** 10.1093/iob/obae002

**Published:** 2024-01-22

**Authors:** A D Rummel, E T Sheehy, E R Schachner, B P Hedrick

**Affiliations:** Department of BioSciences, Rice University, Houston, TX 77005, USA; Department of Ecology and Evolutionary Biology, Tulane University, New Orleans, LA 70118, USA; Department of Physiological Sciences, College of Veterinary Medicine, University of Florida, Gainesville, FL 32603, USA; Department of Biomedical Sciences, College of Veterinary Medicine, Cornell University, Ithaca, NY 14853, USA

## Abstract

Geometric morphometrics has had a profound impact on our understanding of morphological evolution. However, factors such as sample size and the views and elements selected for two-dimensional geometric morphometric (2DGM) analyses, which are often dictated by specimen availability and time rather than study design, may affect the outcomes of those analyses. Leveraging large intraspecific sample sizes (*n* > 70) for two bat species, *Lasiurus borealis* and *Nycticeius humeralis*, we evaluate the impact of sample size on calculations of mean shape, shape variance, and centroid size. Additionally, we assessed the concordance of multiple skull 2D views with one another and characterized morphological variation in skull shape in *L. borealis* and *N. humeralis*, as well as a closely related species, *Lasiurus seminolus*. Given that *L. seminolus* is a morphologically cryptic species with *L. borealis*, we assessed whether differences in skull shape and in 2DGM approach would allow species discrimination. We found that reducing sample size impacted mean shape and increased shape variance, that shape differences were not consistent across views or skull elements, and that trends shown by the views and elements were not all strongly associated with one another. Further, we found that *L. borealis* and *L. seminolus* were statistically different in shape using 2DGM in all views and elements. These results underscore the importance of selecting appropriate sample sizes, 2D views, and elements based on the hypothesis being tested. While there is likely not a generalizable sample size or 2D view that can be employed given the wide variety of research questions and systems evaluated using 2DGM, a generalizable solution to issues with 2DGM presented here is to run preliminary analyses using multiple views, elements, and sample sizes, thus ensuring robust conclusions.

## Portuguese abstract

A morfometria geométrica teve um impacto profundo na compreensão da evolução morfológica. No entanto, fatores como o tamanho amostral, vista anatômica e os elementos selecionados para as análises de morfometria geométricas bidimensionais (MG2D), que geralmente são determinados pela disponibilidade de espécimes e de tempo ao invés do design de estudo, podem afetar os resultados dessas análises. Utilizando grandes tamanhos de amostra intraespecífica (n > 70) em duas espécies de morcegos, *Lasiurus borealis* e *Nycticeius humeralis*, avaliamos o impacto do tamanho amostral nos cálculos da média e da variância da forma, e do tamanho do centroide. Adicionalmente, avaliamos a concordância entre vários planos 2D do crânio e caracterizamos a variação morfológica da forma cranial em *L. borealis* e *N. humeralis*, bem como em uma outra espécie proximamente relacionada*, Lasiurus seminolus*. Dado que *L. seminolus* é uma espécie morfologicamente críptica em relação a *L. borealis*, avaliamos se as diferenças no formato do crânio e na abordagem de MG2D utilizada permitiriam discriminar as espécies. Descobrimos que a redução do tamanho da amostra impactou o formato médio e aumentou a variância da forma, que as diferenças na forma não foram consistentes entre as vistas ou elementos cranianos, e que as tendências apresentadas pelos planos anatômicos e pelos elementos não foram fortemente associadas umas às outras. Adicionalmente, constatamos que as formas de *L. borealis* e *L. seminolus*, quantificadas por MG2D, foram estatisticamente diferentes em todas as vistas e elementos. Esses resultados enfatizam a importância em selecionar tamanhos amostrais, vistas 2D e elementos anatômicos adequados, com base na hipótese testada. Devido à grande diversidade de perguntas de pesquisa e de sistemas avaliados com MG2D, provavelmente não exista um tamanho amostral ou vista 2D que possam ser aplicados de forma generalizada. No entanto, uma solução geral para os problemas envolvendo MG2D aqui apresentados é conduzir análises preliminares utilizando várias vistas anatômicas, elementos e tamanhos amostrais, garantindo assim conclusões mais robustas.

## Introduction

Geometric morphometrics (GM) is a commonly used and valuable method for characterizing biological shape in a statistically rigorous, coordinate-based framework (Bookstein 1991; Slice 2007; Zelditch et al. 2012). GM is often capable of discriminating closely related taxa, which makes it an excellent method for distinguishing intraspecific and interspecific morphological differences (Cordeiro-Estrela et al. 2006; Sztencel-Jabłonka et al. 2009; Gabelaia et al. 2018; Hedrick et al. 2022) as well as analyzing larger scale macroevolutionary trends (Stayton 2005; Mongiardino Koch et al. 2017; Orbach et al. 2018; Orkney et al. 2021). GM techniques are commonly split into either two-dimensional (2D) image-based GM or three-dimensional (3D) GM. Each method has advantages and disadvantages (Ford et al. 2023; Hedrick 2023), but a primary advantage of 2DGM is that it is relatively inexpensive and possible to do within natural history collections themselves, thus not requiring specimen loans. When designing a 2DGM analysis for assessing differences in morphology among closely related taxa, workers must make decisions related to landmark number and placement, intraspecific sample size for determining intraspecific mean shape and shape variance (Cardini et al. 2015, 2021; Cardini and Elton 2007), which element to evaluate, as well as which view or views should be used to characterize shape (e.g., differing views of the skull). Increasing intraspecific sample size and doing GM on multiple elements and views dramatically increases the data collection and analysis time for a given project. Understanding how sample size and 2D view affect estimates of shape is critically important, especially when analyzing closely related species that may have small overall differences in shape, or when planning a study in which time or available specimens are limited.

Vespertilionid bats are the most speciose family of bats (Shi and Rabosky 2015) and are found in a variety of environments on every continent except Antarctica. GM has been commonly employed by evolutionary biologists interested in bat evolution to answer a variety of questions. For example, 2D and 3DGM have been used to address questions of integration and evolvability (Santana and Lofgren 2013; Sorensen et al. 2014; Hedrick et al. 2020b), dietary divergence (Santana and Cheung 2016; Arbour et al. 2019), development (Camacho et al. 2019), and macroevolution (Rojas et al. 2022; Mutumi et al. 2023), among many others. To assess how sample size, skull element, and 2D view impact interpretations using 2DGM, we examine three species of vespertilionid: *Lasiurus borealis, Lasiurus seminolus*, and *Nycticeius humeralis*. Three commonly used skull views from two elements (cranium and mandible) were chosen to assess the impact of 2D view and element choice on analysis: lateral cranial view, ventral cranial view, and lateral mandibular view. Additionally, we evaluate whether two closely related species of lasiurid bat, *L. borealis* and *L. seminolus*, can be distinguished using 2DGM and whether these results are concordant across different elements and views.

Specifically, we evaluate two sets of related questions for these three species. First, using large intraspecific sample sizes for *L. borealis* (*n* = 72) and *N. humeralis* (*n* = 81), we evaluate estimates of mean centroid size (a measure of size that is by definition independent of shape) and mean shape (a measure of biological shape in which all specimens have been standardized to remove effects of size, rotation, and translation) to address the following questions: (1) How does sample size impact estimates of mean centroid size? (2) How does sample size impact mean shape estimates and mean shape variance in GM analyses? We predict that centroid size will not be substantially impacted by sample size (i.e., it can be accurately determined with small sample sizes), as suggested by previous work (e.g., Cardini et al. 2015). Next, we predict that distance from the true mean and mean shape variance will increase with decreasing sample size (Cardini and Elton 2007). Conversely, as the number of samples decreases, we predict less morphological shape disparity will be captured. Second, we evaluate how the chosen 2D view and skull element choice impact biological conclusions from our data: (3) Are different views and elements correlated with one another, suggesting common trends? (4) Are sexual shape dimorphism (SShD) and sexual size dimorphism (SSD) present in our focal species, and if so, are they found consistently for all views and elements? (5) Is it possible to discriminate two closely related lasiurid species using 2DGM and are these results consistent across views and elements? Given that both elements of the skull, the cranium and the mandible, must function together in food processing, and that different views of the same element, such as the lateral and ventral cranium, represent the same structure in 3D, it is often assumed that 2DGM studies utilizing different views and cranial elements would generate concordant results. We predict that different intraspecific views (lateral cranium, ventral cranium, and lateral mandible) will be significantly correlated with one another. Additionally, we predict that SShD and SSD will be consistently found across all views and that SSD will be significant, given that female bats are generally larger than males, which is likely related to the fact that female bats carry increased loads during pregnancy and while carrying pups (Myers 1978). Finally, we predict that *L. borealis* and *L. seminolus* will be discriminable from one another across all views and both the cranium and mandible. These data will build on our understanding of how data collection and study design choices impact 2DGM analyses broadly.

## Methods

### Materials and initial analyses

Crania and mandibulae from *L. borealis* (males: *n* = 24; females: *n* = 48), *L. seminolus* (males: *n* = 10; females: *n* = 12), and *N. humeralis* (males: *n* = 42; females: *n* = 39) were photographed at the Louisiana State University Museum of Natural Sciences (LSUMZ) with a Canon EOS 70D using an EF-S 60 mm macro lens. The camera was mounted on a photostand to ensure the same angle was used in each picture. The crania were photographed in lateral and ventral views, while the mandibulae were photographed in lateral view with the long axis of the mandible parallel to the lens of the camera. All photographs and specimens were mounted by the senior author to ensure consistency and reduce imaging errors. Specimens came from across Louisiana and western Mississippi (see Supplementary Table S1 for locality information).

Skulls were landmarked and semi-landmarked in tpsDIG2 (Rohlf 2006) for each data subset (each view). Landmarks represent homologous anatomical loci, while semi-landmarks represent homologous curves (Zelditch et al. 2012; Goswami et al. 2019). Each landmark was chosen with respect to anatomical characters that were reproducible across the three species. Semi-landmarks were defined by the equidistant division of curves drawn on the perimeter of cranial and mandibular contours (Goswami et al. 2019). Fourteen landmarks and one semi-landmark curve consisting of fifteen semi-landmarks were digitized for the lateral cranium data subset; nineteen landmarks and one semi-landmark curve consisting of six semi-landmarks were digitized for the ventral cranium data subset; and ten landmarks and three semi-landmark curves consisting of six, six, and eighteen semi-landmarks were digitized for the mandible data subset (Supplementary Fig. S1, Supplementary Table S2). The ventral cranium data subset was represented with landmarks on only one half of the crania as asymmetry was not a factor considered in these analyses. All landmarking was done by the E.T.H. to eliminate inter-observer error and was checked by B.P.H. to ensure consistency. In the small number of cases where inconsistency was uncovered, specimens were re-landmarked by the E.T.H.

Landmarks were imported into R v. 4.2.2 (R Core Team 2022) and opened in *geomorph* v. 4.0.5 (Adams et al. 2022; Baken et al. 2021). Each data subset was separately subjected to Generalized Procrustes Analysis (GPA) and semi-landmarks were slid according to the bending energy criterion (Bookstein 1991; Perez et al. 2006; Zelditch et al. 2012). GPA translates, rescales, and rotates each respective landmark configuration into the same shape space. To analyze overall shape trends in morphospace, each data subset was subjected to principal component analysis (PCA) for all three species separately (intraspecific analyses) and again with all species included (interspecific analyses). PCAs were performed in *geomorph*, where principal components (PCs) that represented greater than 10% of total shape variation were examined. Size was represented by log10-transformed centroid size and each individual data subset's corresponding centroid sizes were used in analyses (e.g., lateral shape data used the centroid size derived from the lateral data subset).

### Effects of sample size on the evaluation of centroid size and mean shape

To assess how intraspecific sample size impacts centroid size, mean shape estimations, and morphospace occupation in GM analyses, we randomly subsampled our data using the sample function in R (R Core Team 2022) into five bins with decreasing sample sizes (100, 75, 50, 25, and 10%) for both *L. borealis* (*n* = 72) and *N. humeralis* (*n* = 81) for each of the three data subsets (lateral cranium, ventral cranium, and lateral mandible). The mean shape and mean centroid size for the 100% bin represented the “true” mean of our sample, which is itself a subsample of the mean of all possible samples. For each bin, we permuted the subsampling 1000 times with specimens randomly attributed to each bin such that they represented a subsample of the data at the desired fraction of the full sample size. We set a seed for each iteration using the set.seed function in R to generate reproducible random subsamples. Subsamples within each bin were then compared with the “true” mean of our sample. For centroid size, we calculated the mean centroid sizes for each randomly sampled subsample, and then compared them to the mean centroid size of the 100% bin. We did this by running an ANOVA (e.g., centroid size ∼ subsample percentage) with a Tukey posthoc test to evaluate significant differences in mean centroid sizes between bins across the 1000 iterations, for each data subset. For mean shape, we generated mean shapes for each data subsample by running GPA and then the mshape function in *geomorph*. In this case, all 1000 permutations for mean shapes for the 100% bin were identical to one other and mean shapes for reduced data subsamples were all different from one another based on the specimens randomly attributed to each individual subsample.

Comparisons across data bins for mean shape were done in two ways. First, we calculated the Procrustes distance between the mean shape of the 100% bin and each permuted subsample's mean shape. We ran an ANOVA with a Tukey posthoc test to evaluate significant differences between data bins. Second, we plotted mean shapes in principal component morphospace using convex hulls (5 bins with 1000 permutations each) to visualize how variance in mean shape changed with decreasing sample size. We then used a Procrustes ANOVA of the subsampled shape data and bins (100, 75, 50, 25, and 10%, e.g., Procrustes distance ∼ subsample percentage) using procD.lm with test.type = “var” in *geomorph* and ran pairwise comparisons in *RRPP* v. 1.3.1 to determine significant differences in variance between data subsamples (Collyer and Adams 2018, 2021). For five randomly selected iterations of the 10% bin, we generated deformation grids along PC1 to compare with the complete dataset (100% of the samples) to visually assess whether there were qualitative differences in the primary shape trends along PC1 resulting from reduced sample size.

### Effects of view and element on 2DGM analyses

To examine the association between different 2D data subsets, we used partial least squares (PLS) analyses comparing data subsets (e.g., lateral cranium shape ∼ lateral mandibular shape) for both *L. borealis* and *N. humeralis*. This was done using the two.b.pls function in *geomorph*. The strength of correlation between datasets was represented by the rPLS coefficient and significance was assessed via 1000 iterations. An rPLS value close to 1 indicates a strong association, while a value close to zero indicates a weak association.

Finally, we assessed whether two biological questions were impacted by the view and element chosen: whether there is any variability in intraspecific biological signature across the three studied species based on view/element and whether it is possible to distinguish *L. borealis* from *L. seminolus* using 2DGM. We evaluated whether SShD and SSD were present in our three bat species (*L. borealis, L. seminolus*, and *N. humeralis*) and whether significance differed based on view/element. We performed PCAs for each data subset for each species. By visualizing each species’ distribution in morphospace separately, we were able to evaluate overall shape trends by data subset, distinguishing males and females. Following visualization, we ran a Procrustes ANOVA for each data subset for each of our three species individually to examine SShD. Procrustes ANOVAs were run in *geomorph* using the procD.lm function (shape ∼ size + sex). This totaled nine separate SShD Procrustes ANOVAs (three data subsets per each of the three species). Additional ANOVAs were run for each species to examine SSD using the basic stats package in R (size ∼ sex) for each species dataset. Differences in size for each sex were plotted using violin plots in *ggplot2*. For *L. borealis*, in which the female specimens outnumbered the male specimens, we evened the sample sizes by choosing a random subset of the female specimens equal to the males for the analyses, iterating the random subsampling 1000 times, and in each iteration assessing sexual size and shape dimorphism as described above.

To evaluate differences in the two lasiurid species, PC morphospaces were evaluated for overarching trends in each data subset. We then assessed whether shape could significantly parse the two species after factoring out size (e.g., shape ∼ size + species) for each of the three data subsets using Procrustes ANOVAs. We additionally evaluated differences in size between the two species (e.g., size ∼ species) using ANOVAs.

## Results

### Effects of sample size on 2DGM analyses

**Fig. 1:**
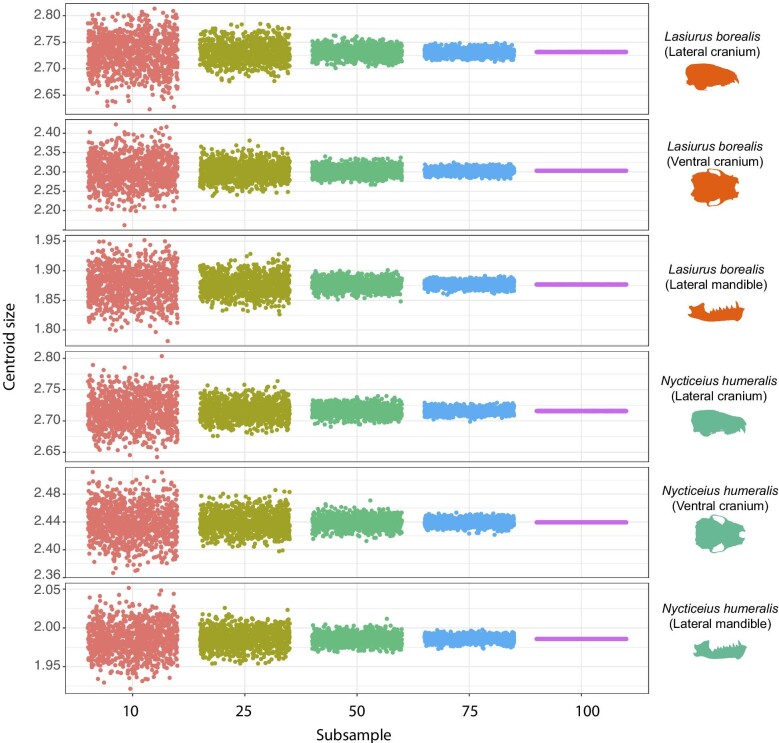
Centroid size calculated for subsampled bins containing 75, 50, 25, and 10% of the data, permuted 1000 times, as well as the true mean centroid size as calculated from 100% of the data for each of the three data subsets (lateral cranium, ventral cranium, lateral mandible) for *L. borealis* and *N. humeralis.* The full dataset for *L. borealis* includes 72 specimens and the *N. humeralis* includes 81 specimens.

We found that there were no significant differences in centroid size among any size bin, including the “true” centroid size calculated from 100% of the data, for any of the data subsets in either *L. borealis* or *N. humeralis* (Fig. 1, Supplementary Table S3). The mean error in calculated centroid size for each of the size bins (75, 50, 25, and 10%) across the 1000 permutations was low, ranging from 0.01 in the 10% subsamples to 0.002 in the 75% subsample (Fig. 1).

**Fig. 2:**
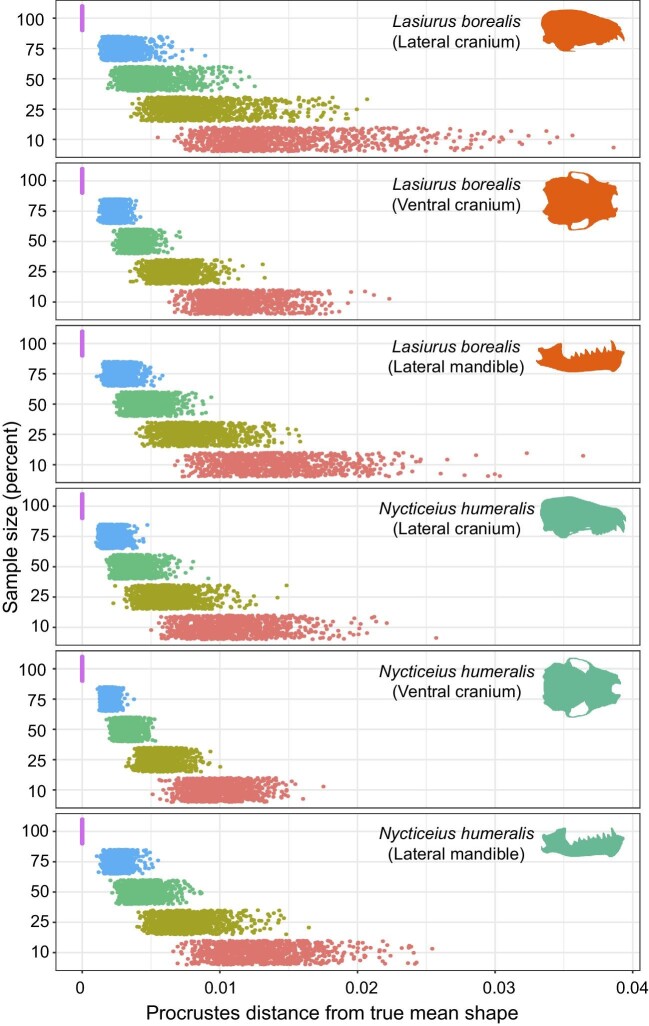
Procrustes distances from the true mean (100% of the sample) across subsampled bins containing 75, 50, 25, and 10% of the data, permuted 1000 times, for each of the three data subsets (lateral cranium, ventral cranium, lateral mandible) for *L. borealis* and *N. humeralis.* The full dataset for *L. borealis* includes 72 specimens and the *N. humeralis* includes 81 specimens.

**Fig. 3:**
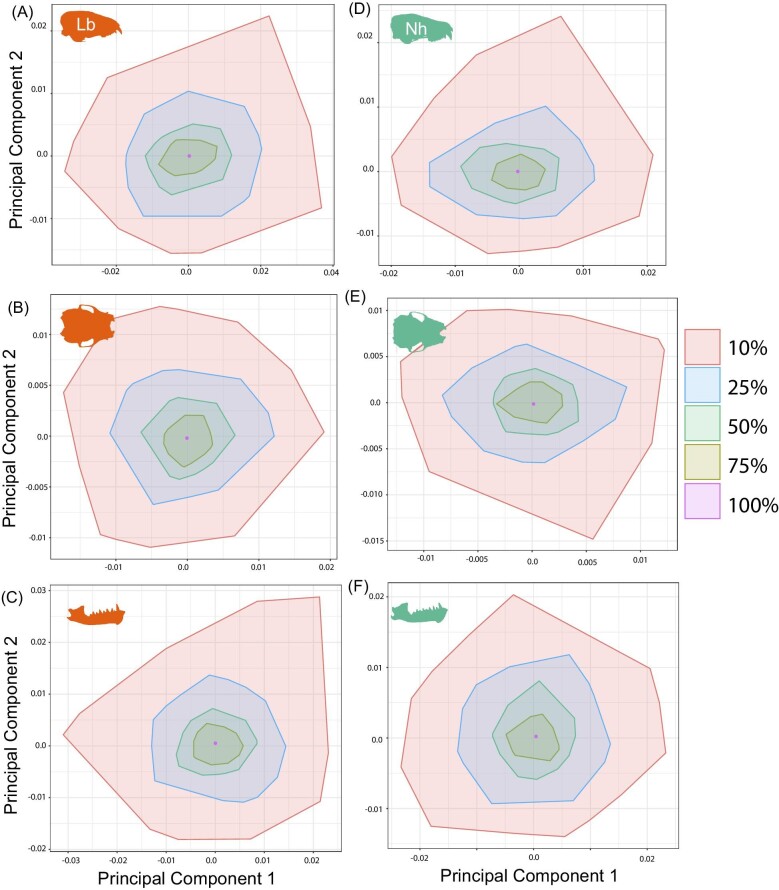
Mean shapes in principal component morphospace relative to the true mean shape, visualized using convex hulls. Each hull represents a subsample containing mean shapes generated using 75, 50, 25, and 10% of the data, with 1000 permutations each, to visualize how variance in mean shape changes with decreasing sample size.

We found that the Procrustes distance between the mean shape subsamples for each bin and the mean shape of the 100% bin was significantly different across all three data subsets for both species (Supplementary Table S4, Fig. 2). We also generally found significant differences in dispersion around the mean for each subsample for all three data subsets in both taxa (Fig. 3, Supplementary Table S5). The exceptions were the lateral cranium data subset of *L. borealis* and *N. humeralis*, as well as the mandible data subset for *L. borealis*, which did not have significant differences in the variance of mean shape between the 75 and 100% bins. These differences in shape variance are visualized in a morphospace, which illustrates the increasing deviation from the 100% sample mean shape with successively decreasing samples (Fig. 3). In the five iterations for which we generated shape grids for the 10% sample size permutation, morphospace occupation along the primary axis of shape change was not qualitatively different from the full sample for either species (Supplementary Fig. S2). Therefore, qualitative interpretations of the primary axis of shape change in the data are unlikely to be impacted by reducing sample size from 70 to 80 individuals to only 7–8 individuals.

### Effects of view and element on 2DGM

**Fig. 4:**
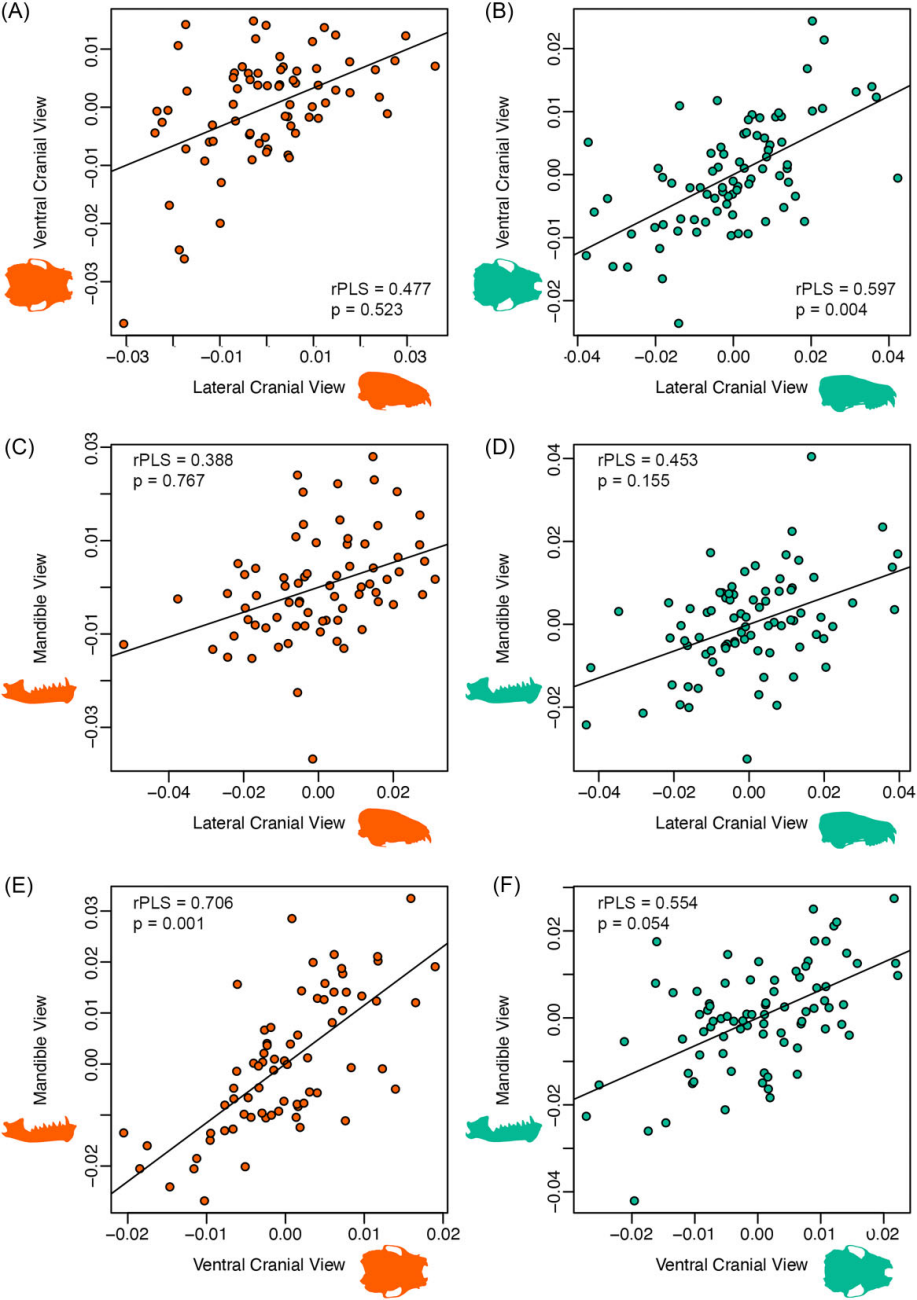
Partial least squares analyses testing for association of views and skull elements for *L. borealis* (left) and *N. humeralis* (right). (A, B) Lateral cranium view compared to ventral cranium view. (C, D) Lateral cranium view compared to mandible view. (E, F) Ventral cranium view compared to mandible view. Reported in each plot are rPLS correlation coefficient between blocks and p-values based on 1000 permutations.

The lateral cranium, ventral cranium, and lateral mandible data subsets were not all strongly associated with one another for either *L. borealis* or *N. humeralis* (Fig. 4). For *L. borealis*, lateral cranium view and ventral cranium view (r-PLS = 0.477, *P* = 0.523) and lateral cranium view and mandible view (r-PLS = 0.388, *P* = 0.767) were not significantly associated with one another. Only the ventral cranium view and mandible view were significantly associated (r-PLS = 0.706, *P* < 0.001). Similar trends were found for *N. humeralis*, where only the lateral cranium view and ventral cranium view were significantly associated (r-PLS = 0.597, *P* = 0.004). Neither lateral cranium view and mandible view (r-PLS = 0.453, *P* = 0.155) nor ventral cranium view and mandible view (r-PLS = 0.554, *P* = 0.054) were significantly associated (Supplementary Table S6).

**Fig. 5:**
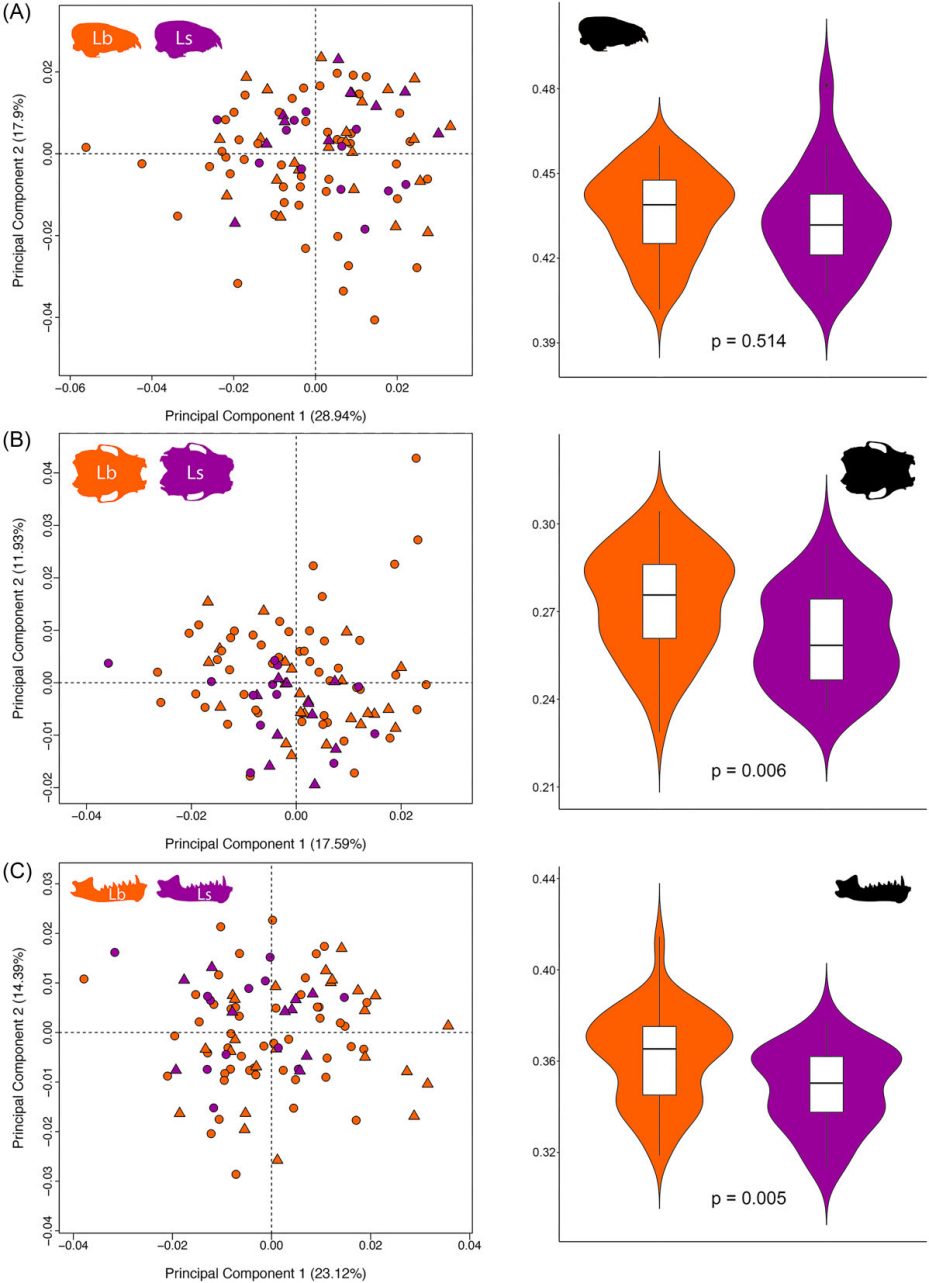
Principal component analyses and violin plots showing differences in shape and size (represented using log10-transformed centroid size) for *Lasiurus borealis* (orange) and *L. seminolus* (purple) in (A) lateral cranial view, (B) ventral cranial view, and (C) lateral mandible view. Circles are females, triangles are males.

The lack of correspondence between views and elements is reflected by biological differences explored in intraspecific analyses for SShD and SSD (PCAs, Supplementary Tables S7–S9). For example, there was significant SShD for some views but not others, generally accounting for small proportions of total variance in cases where it was significant. In *L. seminolus*, only the ventral cranium view had significant SShD (*R*^2^ = 0.08, *P* = 0.03) after accounting for size (Supplementary Table S11). We did not observe significant SShD in any of the *N. humeralis* skull configurations after accounting for size (Supplementary Table S12). SSD was significant in all data subsets for all species with the female being the larger sex. For *L. borealis*, with all male and female specimens included in the analysis, significant SShD was found in the lateral cranium view (*R*^2^ = 0.035, *P* = 0.02) and the ventral cranium view (*R*^2^ = 0.032, *P* = 0.01) after accounting for size, but not the mandible (*R*^2^ = 0.01, *P* = 0.65) (Supplementary Table S10). However, when we randomly subsampled the females such that males and females were equal, we found significant SShD in the lateral cranium in 361 of 1000 permutations, and in the ventral cranium, 526 of 1000 permutations. When comparing the two lasiurid species, we found substantial morphospace overlap for all three data subsets (Fig. 5; Supplementary Table S13). However, both species were found to be significantly different in both shape and size for all three data subsets (except for centroid size in lateral cranial view) (Supplementary Table S14).

## Discussion

### Impacts of sample size on two-dimensional geometric morphometric analyses

Sample size is a key consideration when designing a comparative morphological study. In many cases, sample size is limited by what is available for study in museum collections (Cardini and Elton 2007). This can range from many available specimens for more common or local species to a limited number of specimens for rare, endangered taxa. Knowing the number of specimens required to characterize the mean shape of a taxon is desirable for a range of morphological analyses that utilize GM. The proliferation of phylogenetic comparative methods (PCMs) over the last decade, which often require one individual per tip on a phylogeny, makes the determination of mean shape important as well (see Cornwell and Nakagawa 2017 for a recent review on PCMs). In this case, mean shapes are often based on a set of specimens to account for individual variability in a sample (e.g., Sherratt et al. 2014; Hedrick and Dumont 2018). However, the number of samples utilized to generate those mean shapes may have a significant impact on the resultant mean shape (Figs. 2, 3; Cardini and Elton 2007; Cardini et al. 2015) and thus may impact interspecific comparisons in study designs controlling for phylogeny with PCMs.

Using our sample sizes of >70 specimens each of *L. borealis* and *N. humeralis*, we found that both the shape distance from the true mean of our sample (100% of our sample) and the mean shape variance around the true mean increased with progressively decreased sample sizes (Figs. 2, 3), while estimated centroid size was not significantly affected by sample size (Fig. 1, Supplementary Table S3). This suggests that samples of insufficient number may not accurately capture mean shape. Mean shape was indeed significantly different across all bins (Fig. 2), and mean variance differed between almost all bins (Supplementary Table S5, Fig. 3). However, actual differences in shape visualized as thin plate spline grids from one side of the shape space to the other border on imperceptible (Supplementary Fig. S2). As such, determination of an appropriate sample size should be study-specific. In cases where very similar species are being compared (Sztencel-Jabłonka et al. 2009; Calahorra-Oliart et al. 2021) or when a study is examining cryptic sexual dimorphism (Valenzuela et al. 2004) or intraspecific geographic variation (Marchán-Rivadeneira et al. 2010; Hedrick 2021), large sample sizes will be required since mean shape and mean shape variance differences may be important in distinguishing similar groups.

Intraspecific differences in mean shape may be related to sexual shape dimorphism, which is common in bats and many other species (Myers 1978; Hood 2000; Hedrick 2021; Ospina-Garcés et al. 2021). Although we found that SShD accounted for a small proportion of shape variation in the species included in our study, including specimens of only one sex in species with more substantial SShD would bias mean shape estimates. In cases where SShD may be present, samples should be evaluated for SShD by first running a split-sex analysis (Cardini et al. 2015). Otherwise, mean shapes that are derived from a combination of males and females may be halfway between male and female shapes and may not exist in nature. Shape may also vary across geographic locations. For example, seasonality, precipitation, and temperature variation have been connected to the geographic variation in skull shape present across the range of the phyllostomid bat *Artibeus lituratus* (Marchán-Rivadeneira et al. 2010). Historical specimens present an opportunity to track size and shape changes intraspecifically across time (Hedrick et al. 2020a), with some studies suggesting changes in skull size and/or shape in historical specimens relative to modern specimens (Tomassini et al. 2014; Yue et al. 2020).

### Impacts of view and element choice on two-dimensional geometric morphometric analyses

A major goal of GM analyses is to adequately capture the shape of a structure; a limitation of 2DGM compared with 3DGM is that two dimensions axiomatically limit the dimensions of the specimen that is used to characterize shape, flattening three-dimensional shapes and removing shape data coming from the *z*-axis (Buser et al. 2018; Hedrick et al. 2019; Wasiljew et al. 2020). This issue has been examined with some intensity, with the general conclusion that highly three-dimensional objects should be characterized using 3DGM while the shapes of relatively flattened structures can be adequately captured using 2DGM. Since 3D data collection can take substantially longer and is often more costly than 2D data collection, 2DGM may be preferable (Wasiljew et al. 2020; Ford et al. 2023).

If 2DGM is determined to be an adequate method for capturing specimen data, which view(s) and element(s) should be used to digitize specimens? In crania, common orientations include lateral view and ventral view, with lateral mandible views also often being used (e.g., Hedrick and Dumont 2018). Many studies on vertebrate skulls digitize a single element (either the cranium or the mandible) in a single view, given that multiple views or elements can double or triple data collection time and may offer limited improvements in the ability to address hypotheses. Because each view represents a different, but sometimes overlapping, set of anatomical features, choosing one view over another may not capture critical axes of variation. To determine whether multiple skull views or elements offer non-complementary shape trends, we assessed correlations between our three separate skull data subsets (lateral cranium, ventral cranium, and lateral mandible) for *L. borealis* and *N. humeralis*. Across the three views and two elements in two species, two correlations were significant (lateral cranium vs. ventral cranium in *N. humeralis*, ventral cranium vs. lateral mandible in *L. borealis*), one was marginally significant (ventral cranium vs. lateral mandible in *N. humeralis*), and three were not significant. This suggests that different skull views are not consistent in the shape trends they produce, with different views illustrating different patterns. Further, given that the cranial views represent the same structure, it would be expected that these would be more likely to be correlated with one another than either cranium view with the mandible. However, this was not the case for *L. borealis* and we note relatively high correlations between ventral cranium and mandible views in both *L. borealis* and *N. humeralis*, suggesting that those views may be somewhat complementary even in cases where the ventral cranium and lateral cranium are not (Fig. 4). The skull has a variety of tasks (vision, smell, housing the brain, and food processing) while the mandible is tied solely to food processing (Hedrick and Dumont 2018; Mutumi et al. 2023). Perhaps the relationship between the width of the zygoma and the area of the ramus of the mandible (both attachments for the masseter muscle) is driving this relationship, while the lateral view of the skull is capturing other aspects of cranial function.

Bats generally display substantial intraspecific SSD, whereby females are larger than males (Myers 1978; Sztencel-Jabłonka et al. 2009; Hedrick 2021). We found significant SSD in all three data subsets for all three species examined (Supplementary Tables S8, S11, S12; Supplementary Figs. S3–S5), with females consistently larger than males. Sexual shape dimorphism was less consistent among our three study species. Only the ventral view of the cranium of *L. seminolus*, had significant SShD, and *N. humeralis* did not exhibit significant SShD at all. In *L. borealis*, with all specimens included, we found significant SShD in the lateral and ventral cranium; however, there is significant morphospace overlap (Supplementary Fig. S3) and with equal male and randomly subsetted female sample sizes, SShD was inconsistently significant. This suggests that although differences in shape may be statistically discriminable between males and females, they are qualitatively minor. Recently, Ospina-Garcés and Léon-Paniagua (2021) found that the primary differences between sexes in *N. leporinus* crania are related to sagittal crest shape. Among our three species, only *L. borealis* had significant SShD in lateral view, the view that characterizes the sagittal crest. However, despite significant differences there was no clear clustering of males and females in shape space (Supplementary Fig. S3). Instead, females are dispersed throughout shape space and males cluster in the upper right quadrant of shape space, which aligns with a more prominent sagittal crest. Hayes et al. (2019) found high ordinal dietary richness in female *L. borealis* compared with males. Perhaps this is being reflected in *L. borealis* morphology given the larger expansion of females in shape space. Conversely, Ospina-Garcés and Léon-Paniagua (2021) suggested that the larger cranial crests in *N. leporinus* may allow males to eat larger and more variable prey items than females. Regardless, after factoring out cranial size, SShD made up a very small overall percentage of total shape variance (1–3% in *L. borealis*, 3–8% in *L. seminolus*, and 1–2% in *N. humeralis*) (Supplementary Tables S10–S12). Therefore, while the choice of element and view may have impacted significance, it did not strongly impact the amount of shape variance attributable to sex.

*Lasiurus borealis* and *L. seminolus* are very similar species that are often confused (Wilkins 1987) and have been shown to have similar skull sizes and shapes using linear morphometrics (Lowery Jr. 1975). Laerm et al. (1999) showed that standard body and skull measurements cannot separate the two species and that intraspecific SShD was higher within species in comparison with interspecies differences. GM has been used previously to distinguish two cryptic bat species (*Pipistrellus pipistrellus* and *P. pygmaeus*) that could not be separated with linear measurements (Barlow et al. 1997; Sztencel-Jabłonka et al. 2009). Previous linear morphometric work on *Artibeus* also uncovered high morphological overlap across many species (Marchán-Rivadeneira et al. 2010), while GM analyses were able to uncover statistically significant differences (Hedrick 2021). We found that GM allowed some statistical separation in shape and size between *L. borealis* and *L. seminolus.* Further, this was consistent across views and elements (except for lateral cranial view). However, there was very minor separation between the two *Lasiurus* species in shape space with *L. seminolus* generally plotting within *L. borealis* morphospace (Fig. 5). Given that *L. seminolus* size and shape space lie nearly entirely within *L. borealis* size and shape space, discrimination using 2DGM is likely not possible. *L. borealis* and *L. seminolus* have cranial morphologies consistent with harder prey, such as coelopterans (Freeman 1984; Feldhamer et al. 2009). Given that vespertilionid bats are known to have skull shapes significantly associated with phylogeny (Hedrick and Dumont 2018), these data suggest that phylogeny, as well as dietary ecology, plays a role in shaping the skulls of these closely related and ecologically similar species.

Just as in choosing between 2DGM and 3DGM analyses, the view chosen should match the aim of the study, the specimens used, and the hypotheses that are being tested. For example, Hedrick and Dumont (2018) used 2DGM on bat skulls digitizing specimens in lateral cranium view and lateral mandible view. Ventral view was not used since prior studies had suggested skull height was the most effective metric for distinguishing diet in phyllostomid bats (Dumont et al. 2009; Santana and Dumont 2009; Santana et al. 2010), a primary goal of that study. Despite this, ventral view may have added valuable, perhaps non-complementary information to Hedrick and Dumont’s (2018) study. The ventral view of a mammal skull shows the shape and size of the zygomatic arch, which may also have been relevant to diet. When it is difficult to determine which view may be most relevant to a given question, doing preliminary analyses to assess whether different views are strongly correlated with one another as has been done here is critical prior to collecting and digitizing entire datasets in multiple views. This will save data collection time and lead to more robust results and conclusions.

## Conclusions

Using 2DGM and relatively large intraspecific sample sizes, we evaluated the effect of sample size in cases where morphological variation may be subtle. We found that although mean shape deviated from the “true” mean, and shape variance increased with decreasing sample sizes, qualitative shape differences along the primary axis of variance were negligible. Additionally, we found inconsistent correlation between shape variation in the three 2D views we selected for this study. These results reinforce the importance of determining appropriate sample sizes, 2D views, and skull element before data collection. Conversely, centroid size estimates were not impacted by reducing sample size for any view or element. These results were reinforced when using these data to assess questions of sexual dimorphism in our three bat species whereby significance differed across views and elements for SShD, but not for SSD. We found a consistent significant difference in shape across views and elements between *L. borealis* and *L. seminolus*, but this difference accounted for a very small component of total shape variation and morphospace overlap was substantial. We emphasize that although there may not be a universal rule of thumb for choosing sample size or 2D view given the breadth of study systems and research questions, performing preliminary analyses helps determine what sample sizes are necessary to address a given question and whether different 2D views offer analogous results and can lead to more robust conclusions.

## Supplementary Material

obae002_Supplemental_Files

## Data Availability

Data underlying this article are available in its online supplementary material.
