# Supplementary material for: Sample Size and Geometric Morphometrics Methodology Impact the Evaluation of Morphological Variation
Source: Integr Org Biol. 2024 Jan 22;6(1):obae002. doi: 10.1093/iob/obae002 (PMC10833145; doi:10.1093/iob/obae002)
Supplement: obae002_Supplemental_Files [file obae002_supplemental_files.zip › supplemental figures.docx]

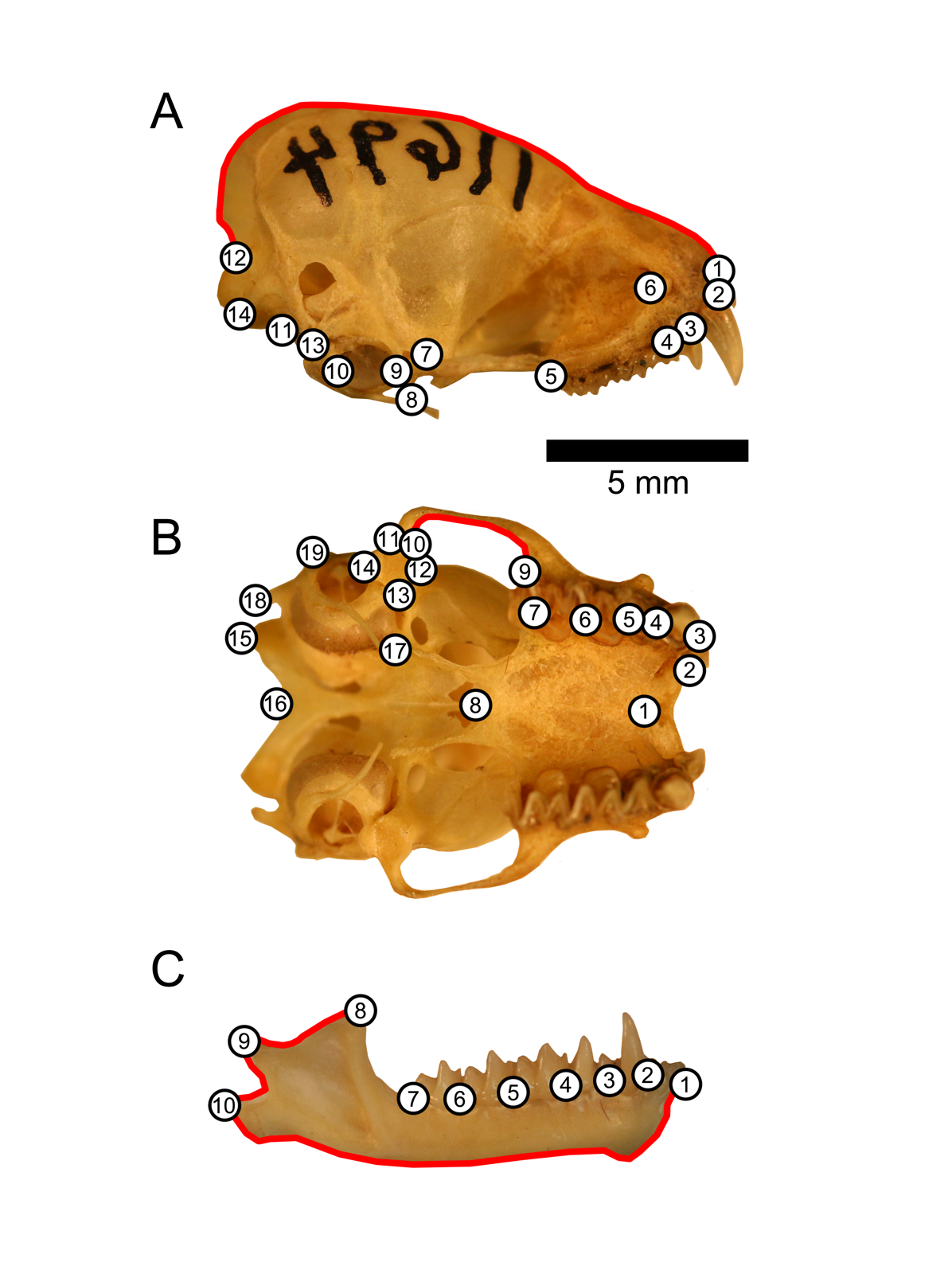


Fig. S1: Landmarking scheme for the (A) lateral cranium view, (B) ventral cranium view, and (C) mandible view used for *L. borealis*, *L. seminolus*, and *N. humeralis*. Landmarks are labeled with descriptions found in Table S1. Red lines represent semi-landmark curves. Scale = 5 mm.


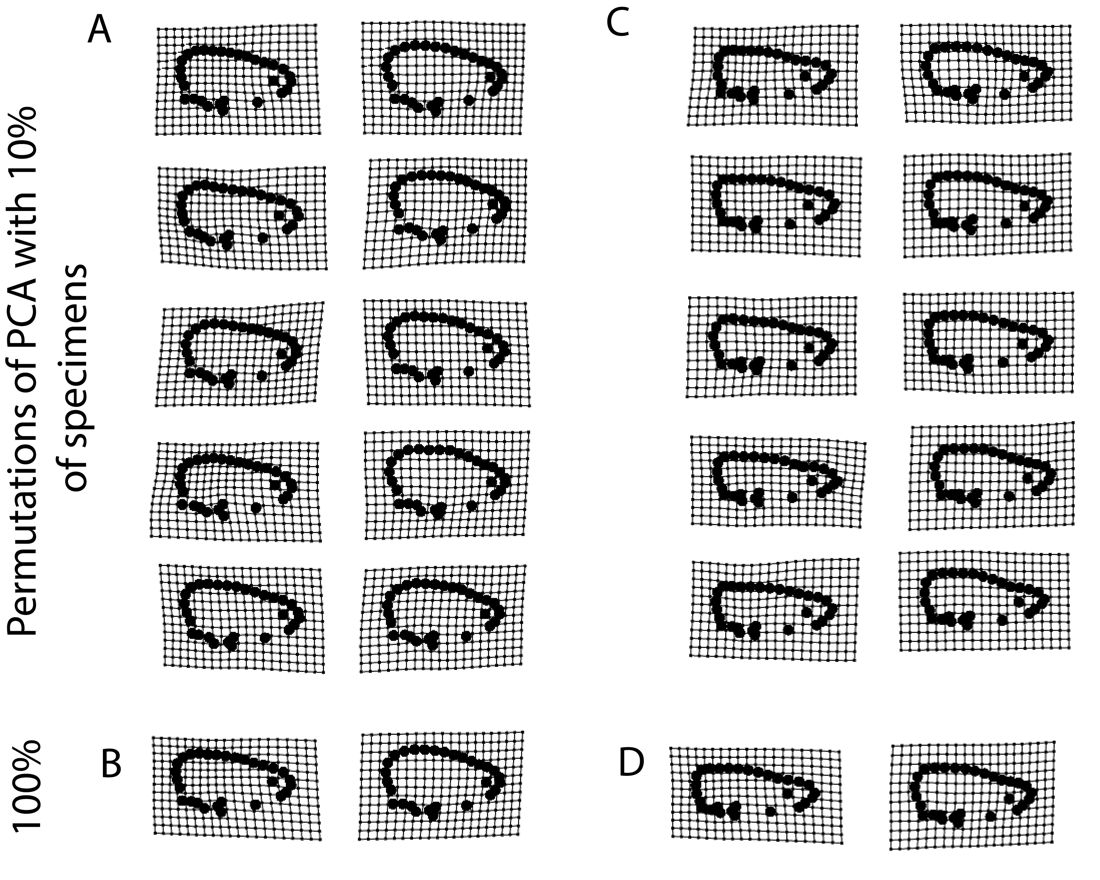


Fig. S2: Assessments of changes to major shape trends as a result of reducing sample size for *L. borealis* (A, B) and *N. humeralis* (C, D). The full dataset for *L. borealis* includes 72 specimens and the *N. humeralis* includes 81 specimens. The reduced datasets with 10% of the specimens include 7 *L. borealis* specimens and 8 *N. humeralis* specimens. (A, C) Major shape trends along principal component 1 for five randomly selected reduced datasets using only 10% of the total number of specimens included in our analysis. (B, D) Major shape trends along principal component 1 for the full dataset.


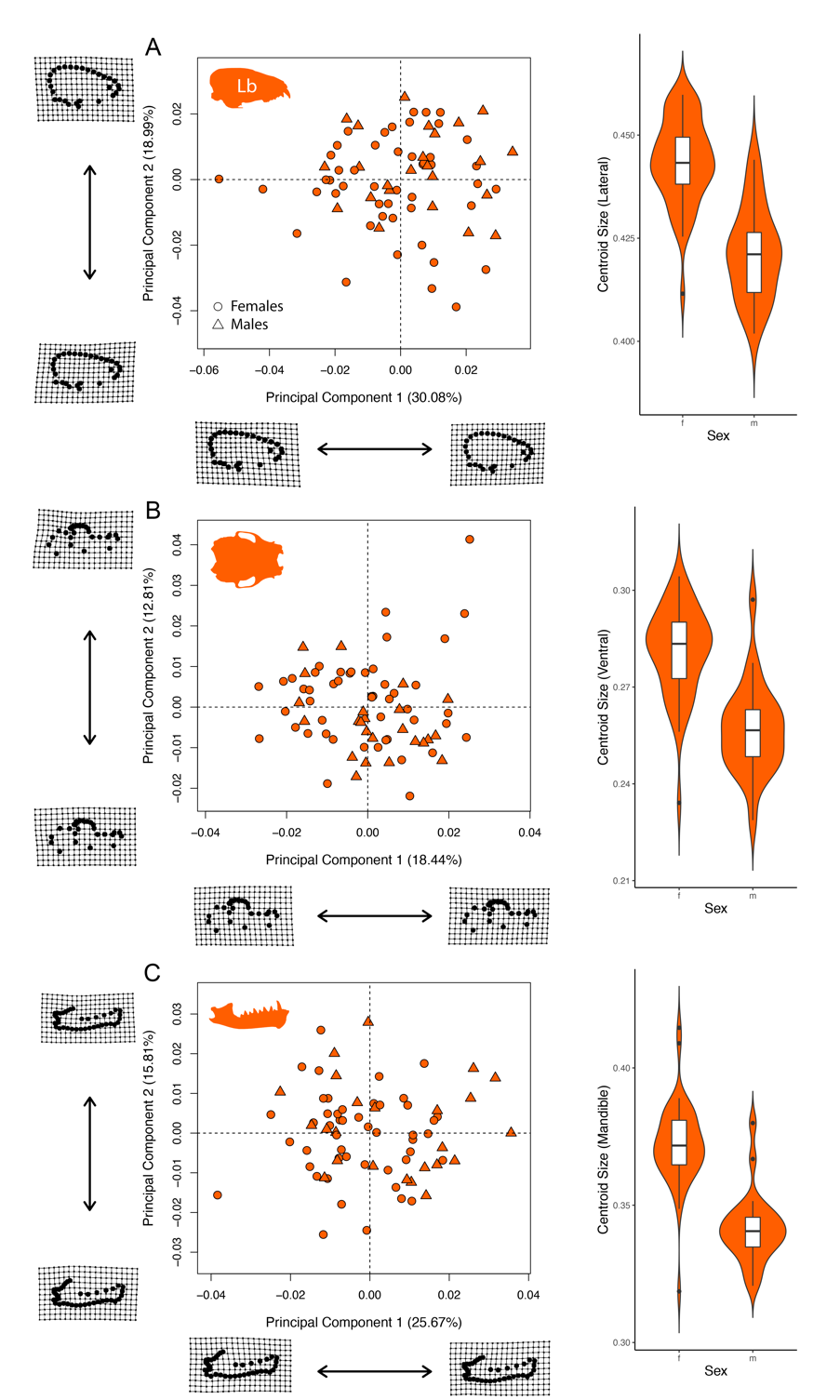


Fig. S3: Shape spaces and size comparisons for all three views for *L. borealis*. Principal component morphospaces showing principal component 1 and 2 for (A) lateral cranium view, (B) ventral cranium view, and (C) mandibular view. Females are represented by circles and males are represented by triangles. Thin plate splines (TPS) show the maximum and minimum shape along the PC1 and PC2 axes (PC1 TPS grids below the plots and PC2 TPS grids to the left of the plots). To the right of each principal component plot is a comparison of log10-transformed centroid sizes for each view visualized as violin plots allowing assessment of sexual size dimorphism. m = males, f = females.


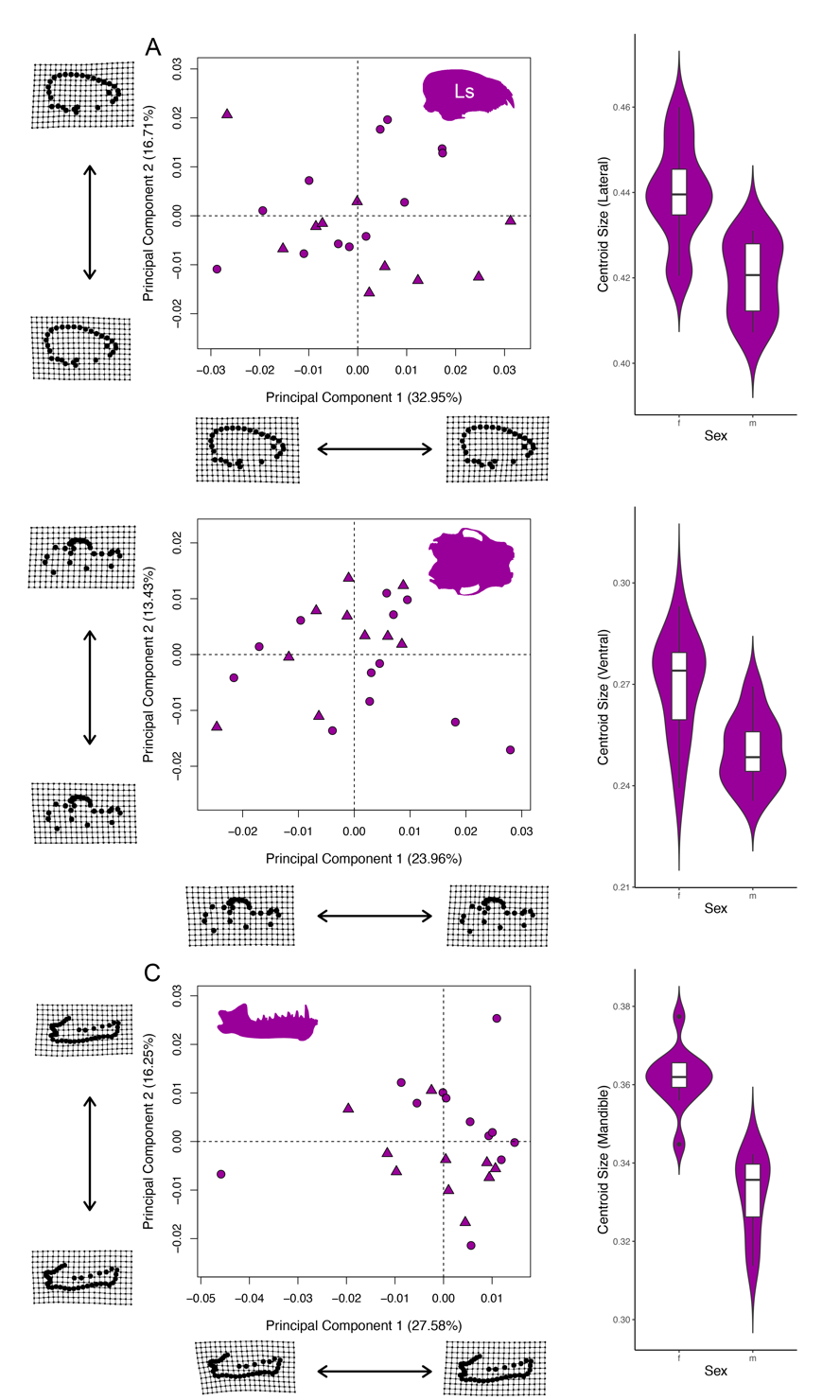


Fig. S4: Shape spaces and size comparisons for all three views for *L. seminolus*. Principal component morphospaces showing principal component 1 and 2 for (A) lateral cranium view, (B) ventral cranium view, and (C) mandibular view. Females are represented by circles and males are represented by triangles. Thin plate splines (TPS) show the maximum and minimum shape along the PC1 and PC2 axes (PC1 TPS grids below the plots and PC2 TPS grids to the left of the plots). To the right of each principal component plot is a comparison of log10-transformed centroid sizes for each view visualized as violin plots allowing assessment of sexual size dimorphism. m = males, f = females.


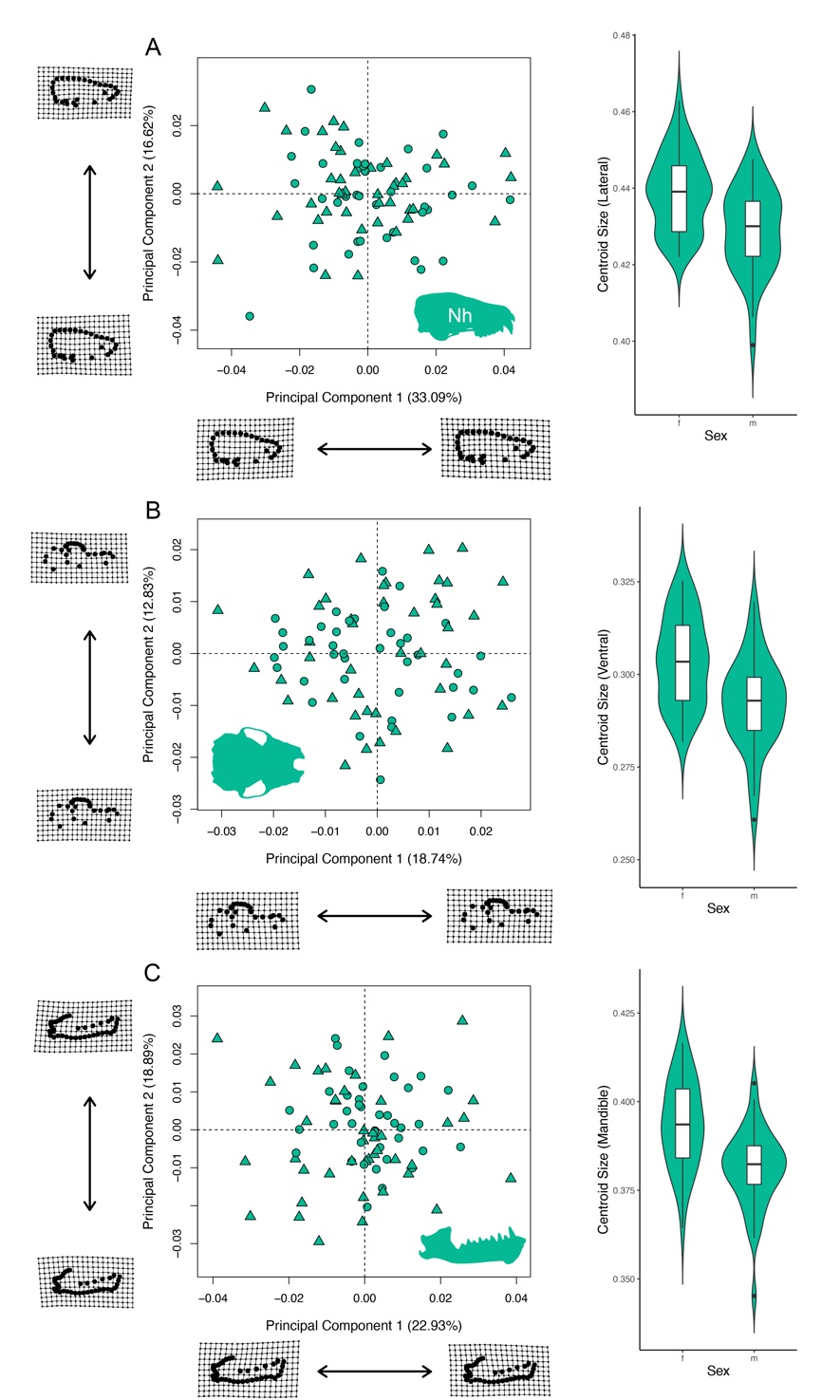


Fig. S5: Shape spaces and size comparisons for all three views for *N. humeralis*. Principal component morphospaces showing principal component 1 and 2 for (A) lateral cranium view, (B) ventral cranium view, and (C) mandibular view. Females are represented by circles and males are represented by triangles. Thin plate splines (TPS) show the maximum and minimum shape along the PC1 and PC2 axes (PC1 TPS grids below the plots and PC2 TPS grids to the left of the plots). To the right of each principal component plot is a comparison of log10-transformed centroid sizes for each view visualized as violin plots allowing assessment of sexual size dimorphism. m = males, f = females.
